# Supplementary material for: Anodic TiO2 Nanotube Layers for Wastewater and Air Treatments: Assessment of Performance Using Sulfamethoxazole Degradation and N2O Reduction
Source: Molecules. 2022 Dec 16;27(24):8959. doi: 10.3390/molecules27248959 (PMC9782093; doi:10.3390/molecules27248959)
Supplement: Supplementary file 1 [file molecules-27-08959-s001.zip › molecules-2081901-supplementary.pdf]

Supplementary Materials

Anodic TiO_2 nanotube layers for wastewater and air treatments: Assessment of performance using sulfamethoxazole degradation and N_2O reduction

Marcel Sihor [1,2], Sridhar Gowrisankaran [1], Alexandr Martaus [2], Martin Motola [1], Gilles Mailhot [3], Marcello Brigante [3] and Olivier Monfort [1,*]

[1] Department of Inorganic Chemistry, Faculty of Natural Sciences, Comenius University Bratislava, Ilkovicova 6, Mlynska Dolina, 84215 Bratislava, Slovakia
[2] Institute of Environmental Technology, CEET, VSB-Technical University of Ostrava, 17. Listopadu 15/2172, 70800 Ostrava-Poruba, Czech Republic
[3] Institut de Chimie de Clermont-Ferrand, Université Clermont Auvergne, CNRS, Clermont Auvergne INP, F-63000 Clermont-Ferrand, France
* Correspondence: monfort1@uniba.sk; Tel.: +421-290142141

Table S1. LC-MS data of SMX degradation by-products

Compound	Rt *(min)*	Exp *[M+H]*$^+$	Theor *[M+H]*$^+$	Δmu	Exp *[M-H]*$^-$	Theo *[M-H]*$^-$	Δmmu	Molecular formula	Proposed Structure
SMX	2.92	254.0592<br>276.0410<br>*[M+Na]*$^+$	254.0594	0.18	252.0440	252.0437	0.29	$C_{10}H_{11}N_3O_3S$	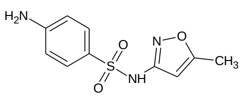
P1	1.84	254.0592	254.0594	0.17				$C_{10}H_{11}N_3O_3S$	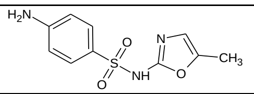
P2	2.74	270.0542	270.0543	0.08	268.393	268.0387	0.69	$C_{10}H_{11}N_3O_4S$	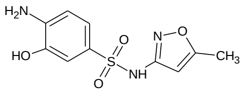
P3	2.21	286.0487	286.0492	0.52	284.0343	284.0336	0.75	$C_{10}H_{11}N_3O_5S$	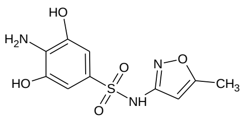
P4	1.00	288.0645	288.0649	0.39	286.0497	286.0492	0.72	$C_{10}H_{13}N_3O_5S$	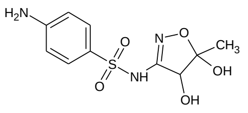
P5	1.92				172.0966	172.0063	0.90	$C_6H_{11}N_3O_5S$	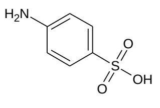
P6	0.87	99.0557	99.0553	0.43				$C_4H_7N_2O$	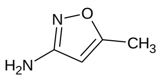
P7	1.04				196.0174	196.0175	0.15	$C_7H_7N_3O_2S$	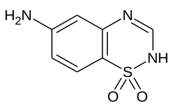

P7	0.86				212.0125	212.0124	0.02	$C_7H_7N_3O_3S$	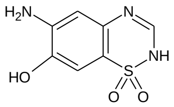
P8	1.76				197.0014	197.0015	0.01	$C_7H_6N_2O_3S$	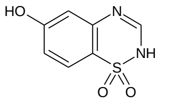

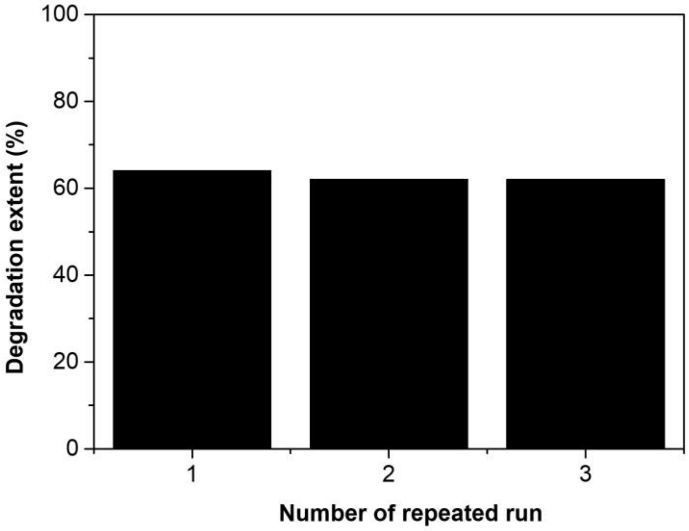

Figure S1. Reusability of sample F20 in SMX degradation after 3 repeated runs.

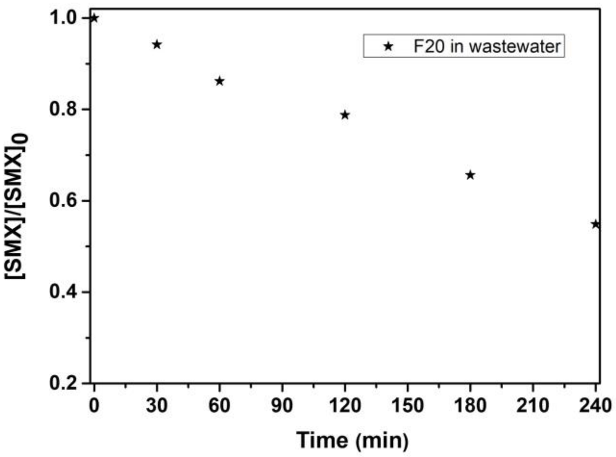

Figure S2. Degradation of SMX in secondary effluents of wastewater treatment plant using F20 under UVA light.
